# Comprehensive analysis of oncogenic signatures and consequent repurposed drugs in *TMPRSS2:ERG* fusion‐positive prostate cancer

**DOI:** 10.1002/ctm2.420

**Published:** 2021-05-13

**Authors:** Jae Won Yun, Sejoon Lee, Sejong Chun, Kwang Woo Lee, Jongsu Kim, Hong Sook Kim

**Affiliations:** ^1^ Veterans Health Service Medical Center Veterans Medical Research Institute Seoul Korea; ^2^ Precision Medicine Center Seoul National University Bundang Hospital Seongnam South Korea; ^3^ Department of Pathology and Translational Medicine Seoul National University Bundang Hospital Seongnam South Korea; ^4^ Department of Laboratory Medicine Chonnam National University Medical School and Hospital Gwangju Korea; ^5^ School of Medicine University of Queensland Brisbane Australia; ^6^ Department of Biological Sciences Sungkyunkwan University Suwon Korea

Dear Editor,

*TMPRSS2:ERG* (TE) fusion occurs in approximately 50% of all prostate cancer cases.^1^ However, details about altered signaling or the difference of gene expression regarding potential therapeutic targets between TE fusion‐positive and negative group is yet to be fully investigated. In this study, we investigated the landscape of molecular signaling and curated potential therapeutic targets in TE fusion‐positive prostate cancers using The Cancer Genome Atlas data. Firstly, we identified 3870 genes in coordination with *ERG* in RNA expression and nine cancer‐related pathways specifically altered in TE fusion‐positive prostate cancer patients. Secondly, we deduced repositionable 55 drugs targeting for TE fusion‐positive prostate cancer from network analysis. Finally, we provided experimental data for six drugs obtained from our in silico analysis and showed sensitivity specific for TE fusion‐positive prostate cancer cell line.

This study is designed as shown in the overview (Figure 1): First, after getting RNA‐seq data and clinical information from broad global data assembly centers firehose (GDAC, https://gdac.broadinstitute.org/), we selected the genes correlated with *ERG* using Pearson correlation test (|R| > 0.3) with the RNA expression level of each gene (Figure S1). Second, pathway analysis was performed using ConsensusPathDB (CPDB, http://consensuspathdb.org/)^2^ and pathways with key altered genes were visualized (Figures 2 and S2‐S4.). Third, potential actionable drugs were inferred through network analysis using Clinical Interpretation of Variants in Cancer (CIViC) database (https://civicdb.org/)^3^ and *ERG* correlated gene list (Figure 3A). Finally, among the actionable drugs inferred, seven drugs were selected, and drug sensitiveness was tested using TE fusion‐positive and negative prostate cancer cell lines (Figure 3B).

**FIGURE 1:**
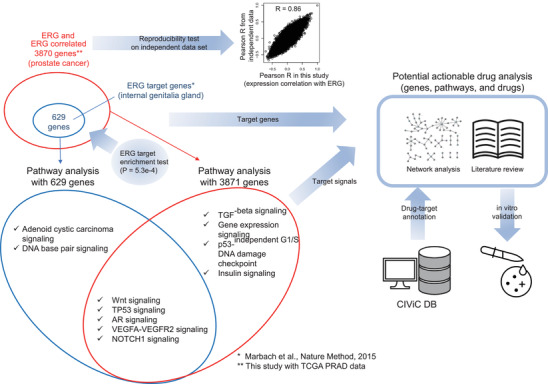
Overview of the analysis in this study. After *ERG*‐correlated genes in RNA level were selected, the ERG target gene enrichment test was performed for validation. Then pathway analysis by over‐representation analysis was performed based on the intersection of *ERG* target genes and *ERG* correlated genes with the *ERG*‐affected genes and altered signals specific for TE fusion‐positive group, an association between anti‐cancer drugs and their actionability for target genes was analyzed through network analysis, literature review, and target gene‐drug annotation

**FIGURE 2:**
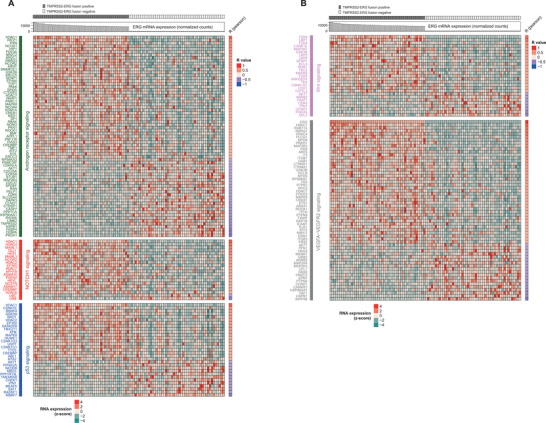
Gene expression heatmap of cancer‐related pathways specifically altered in *TMPRSS2:ERG* (TE) fusion‐positive prostate cancer. Gene expression heatmap of androgen receptor signaling, *NOTCH1* signaling, *p53* signaling (A), *Wnt*‐signaling and *VEGFA*‐*VEGFR2* signaling (B) genes which were upregulated or downregulated specifically in TE fusion‐positive prostate cancers. Rows are representing altered signalings (q‐value < 0.1 in over‐representation analysis) and genes which are correlated with *ERG* in RNA expression (R > 0.3 in Pearson correlation test). Fifty cases with TE fusion and *ERG* upregulation were enrolled in this analysis with 50 fusion‐negative controls

**FIGURE 3:**
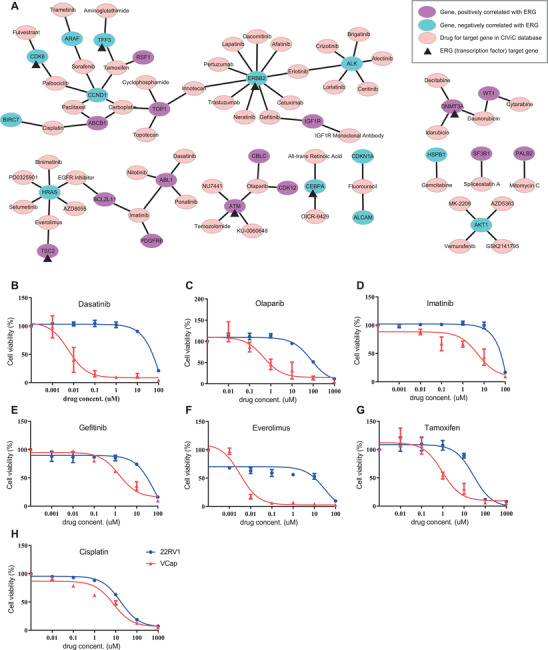
Drug‐target gene network analysis and in vitro drug sensitivity test of candidate drugs specific for *TMPRSS2:ERG* (TE) fusion‐positive prostate cancer. Network analysis was performed among altered genes correlated with *ERG* in expression, and drugs for therapeutic biomarkers (target genes) were selected based on the CIViC database in various cancer types. In drug‐target gene network, some drugs such as olaparib and everolimus are related with at least two potential actionable genes (A). For example, the actionability of irinotecan for *TOP1* expression in TE fusion‐positive group or for *ERBB2* expression in the TE fusion‐negative group could be considered. As for olaparib, its actionability for attenuation of *ATM* and *CDK12* in TE fusion‐negative group could be considered. In vitro drug sensitivity test of candidate drugs selected *by in silico* analysis in TE fusion‐positive and fusion‐negative cell line. VCap cells, TE fusion‐positive cells, showed sensitive to dasatinib, olaparib, imatinib, gefitinib, everolimus, and tamoxifen compared to 22RV1 cells, TE fusion‐negative cells (B‐G). There showed no sensitivity in cisplatin (H)

In overrepresentation analysis for elucidation of target cellular pathways in TE fusion‐positive prostate cancer patients, we identified nine altered signaling pathways, including Wnt signaling, Androgen receptor (*AR*) signaling, gene expression signaling, *VEGFA*‐*VEGFR2* signaling, *p53* signaling, *NOTCH1* signaling, *TGF*‐beta signaling, *p53*‐independent G1/S DNA damage checkpoint, and insulin signaling (Figures 2 and S2‐S4), and confirmed it in validation set except *p53*‐independent G1/S DNA damage checkpoint due to low incidence (Figure S5). Among them, various kinds of *HDAC*s (*HDAC*1, *HDAC*2, *HDAC*4, *HDAC*6, and *HDAC7)* were found to be participating in five signaling pathways (Figures 2 and S2‐S3), suggesting that *ERG* upregulation alters *HDAC*1,2,4,6 and *7*, which can play key roles in prostate cancer signaling. Indeed, we found that *HDAC1* showed the best correlation with *ERG* in RNA expression (R = 0.82). These results are supported by previous study that *ERG* is known to form ESET (*ERG*‐associated protein with a SET domain) with *HDAC1*, and ESET is related with pluripotency and de‐differentiation which is important signaling in cancer.^4^, ^5^ We also identified well established cancer‐specific genes (Table S1) that were associated with TE fusion‐positive cases. In case of AR signaling (Figure 2A), 33 of cancer‐related genes are altered including *EP300*, *CREBBP, CDKN1A, AKT1, CCND1*, CDK6, and *TMPRSS2* genes in TE‐positive group. As consistent with our analysis, different androgen profiles were observed in TE fusion‐positive patients.^6^ In addition, clinical impact of androgen has been introduced, specifically, androgen deprivation therapy showed survival benefit in TE fusion positive prostate.^7^ Wnt signaling was also confirmed to be altered in pathway analysis (Figure 2B). In this pathway, 14 of cancer‐related genes are altered including *TSC2*, *CDH1, TCF7L2*, *AKT1*, *CDK6*, and *CCND1* genes. In addition, alteration of TGF‐beta, *NOTCH1*, *VEGFA‐VEGFR2*, gene expression, and insulin signaling were also listed to be associated with *ERG* expression (Figures 2 and S2‐S3). Intriguingly, although various molecular pathways and associated genes are significantly altered in RNA‐level (Figure 2), clinical phenotype is not significantly different between the TE fusion‐positive and the negative group (Table S2). These results indicate that even if the patient's clinical phenotype is similar, the molecular subtype is different at the molecular level, and therefore the drug target is significantly different, indicating that understanding molecular features in each patient is crucial for cancer therapy.

Next, we examined therapeutic targets and potential actionable drugs through the pathway analysis based on RNA expression and the annotation with drug‐target database. In this analysis, RNA expression of 28 genes, which could be potential therapeutic targets, was observed to have different levels of alteration between TE fusion‐positive and negative groups, and they were involved in various signaling pathway (Table 1). Interestingly, in gene‐drug network analysis, 14 drugs were found to be related with multiple genes among 28 genes which were up‐ or downregulated in TE fusion‐positive group (Figure 3A). To prove whether our *in silico* analysis method is supporting the idea of drug repurposing, we randomly selected seven drugs and performed cell viability test in VCap cells, TE fusion‐positive cells and 22RV1 cells, TE fusion‐negative cells. Dasatinib (targeting *ABL1*), imatinib (targeting *ABL1*, *PDGFRB*, and *BCL2L11*), and olaparib (targeting *CBLC*, *CDK12*, and *ATM*) effectively reduced viability of VCap cells compared to 22RV1 as expected because the expression of these genes targeted by dasatinib, imatinib, and olaparib was positively correlated with ERG expression (Figures 3A and 3B). Gefitinib, targeting *IGF1R* and *ERBB2* inhibited viability of VCap cells compared to 22RV1 cells, and it is thought to be via inhibition of upregulated *IGF1R* rather than downregulated *ERBB2* (Figure 3B). In addition, we tested effect of everolimus, tamoxifen, and cisplatin. Everolimus, tamoxifen, and cisplatin target genes whose expressions are both positively and negatively correlated with ERG expression. Everolimus and tamoxifen decreased Vcap cell viability compared to 22RV1 (Figure 3B). Although cisplatin is one of classical chemotherapeutic agent based on NCCN guideline,^8^ cisplatin does not seem to be specifically effective in TE fusion‐positive prostate cancer cell line in our *in vitro* study (Figure 3B). Cisplatin could target both *BIRC7* and *ABCB1*, and its effect could be offset. We need to further explore the effect of drugs in each target genes and signaling pathway to precisely understand underlying mechanism of each drug. But at least, we here suggest that our systematic *in silico* analysis is proper approach for drug repurposing study.

**TABLE 1:** List of 28 actionable target genes which are correlated with ERG in RNA expression based on the CIViC database

Target gene	Correlation with ERG expression (R value)	Involved pathways	Potential actionable drug for target gene
WT1	0.55	TGF‐beta signaling	Cytarabine, Daunorubicin
IGF1R	0.43	Insulin Signaling	Gefitinib, IGF1R Monoclonal Antibody
DNMT3A	0.43	Gene Expression	Daunorubicin, Decitabine, Idarubicin
TOP1	0.42	Androgen receptor signaling	Carboplatin, Cyclophosphamide, Irinotecan, Topotecan
CBLC	0.42	Insulin Signaling	Olaparib
ATM	0.39	p53 pathway, gene expression, p53‐independent G1/S DNA damage checkpoint	KU‐0060648, NU7441, Olaparib, Temozolomide
BCL2L11	0.38	NA	EGFR Inhibitor, Imatinib
CDK12	0.38	Gene expression	Olaparib
TSC2	0.38	wnt signaling, insulin signaling, gene expression	Everolimus
RSF1	0.37	NA	Tamoxifen
ABCB1	0.34	NA	Carboplatin, Cisplatin, Paclitaxel
PALB2	0.32	NA	Mitomycin C
PDGFRB	0.31	NA	Imatinib
ABL1	0.31	p53 pathway	Dasatinib, Imatinib, Nilotinib, Ponatinib
SF3B1	0.30	Gene expression	Spliceostatin A
ARAF	‐0.31	NA	Sorafenib, Trametinib
ALK	‐0.31	Gene expression	Alectinib, Brigatinib, Ceritinib, Crizotinib, Erlotinib, Lorlatinib
CDKN1A	‐0.31	Androgen receptor signaling, gene expression, NOTCH1 signaling	Fluorouracil
AKT1	‐0.31	p53 pathway, wnt signaling, VEGFA‐VEGFR2 signaling, androgen receptor signaling, insulin signaling, gene expression, NOTCH1 signaling	AZD5363, GSK2141795, MK‐2206, Vemurafenib
HRAS	‐0.32	VEGFA‐VEGFR2 signaling, insulin signaling	AZD8055, Binimetinib, EGFR Inhibitor, Everolimus, PD0325901, Selumetinib
BIRC7	‐0.35	NA	Cisplatin
CDK6	‐0.36	wnt signaling, androgen receptor signaling	Fulvestrant, Palbociclib
CCND1	‐0.40	wnt signaling, VEGFA‐VEGFR2 signaling, androgen receptor signaling, NOTCH1 signaling	Carboplatin, Paclitaxel, Palbociclib, Sorafenib, Tamoxifen
ERBB2	‐0.41	Gene expression	Afatinib, Cetuximab, Dacomitinib, Erlotinib, Gefitinib, Irinotecan, Lapatinib, Neratinib, Pertuzumab, Trastuzumab
ALCAM	‐0.41	NA	Fluorouracil
HSPB1	‐0.47	VEGFA‐VEGFR2 signaling, androgen receptor signaling, gene expression	Gemcitabine
TFF3	‐0.52	NA	Aminoglutethimide, Tamoxifen
CEBPA	‐0.56	Androgen receptor signaling	All‐trans Retinoic Acid, OICR‐9429

Taken together, we provided the portrait of cellular signaling pathways and prioritized therapeutic targets correlated with *ERG* expression in TE fusion‐positive prostate cancer. We believe that this study will further advance precision medicine in prostate cancer treatment.

## Supporting information

Supporting information.

Supporting information.

Supporting information.

Supporting information.

Supporting information.

Supporting information.

Supporting information.

Supporting information.
