# Supplementary material for: Comprehensive analysis of oncogenic signatures and consequent repurposed drugs in TMPRSS2:ERG fusion‐positive prostate cancer
Source: Clin Transl Med. 2021 May 13;11(5):e420. doi: 10.1002/ctm2.420 (PMC8120022; doi:10.1002/ctm2.420)
Supplement: Supplementary file 1 — Supporting information. [file CTM2-11-e420-s004.docx]

**Figure S1. RNA expression values of a randomly selected gene of 100 prostate cancer sample were randomly reordered and their correlation with *ERG* RNA expression.**

The distribution of R values between *ERG* expression values and randomly reordered expression values of randomly selected genes in 100 TCGA samples were calculated. This process was performed repeatably 100,000 times. As a result, the probability of |R|>0.3 is 0.001525 (0.1525%), and it is estimated that only about 30 genes (19927 x 0.001525) of all 19927 genes included in our study are considered as false positives in all genes with |R|>0.3.

**Figure S2. Gene expression heatmap of *TGF*-beta signaling, *p53*-Independant G1/S DNA damage checkpoint signaling, and insulin signaling genes which were up-regulated or down-regulated specifically in *TMPRSS2:ERG* (TE) fusion-positive prostate cancers.**

**Figure S3. Gene expression signaling related genes which were up-regulated or down-regulated specifically in *TMPRSS2:ERG* (TE) fusion-positive prostate cancers.**

**Figure S4. Nine altered signalings in *TMPRSS2:ERG* (TE) fusion positive prostate cancer.**

Key genes correlated with *ERG* in RNA expression (R > 0.3 in Pearson correlation test) were selected and network analysis was performed (top left). Among the gene nodes, nodes with at least two edges to different signaling were selected and visualized (mid down). Gene nodes were described in pink color and signaling nodes in blue color. Various *HDACs* were participating in multiple signalings including *AR* signaling, *NOTCH1* signaling, *TGF*-beta signaling and *VEGFA-VEGFR2* signaling.

**Figure S5. Validation of nine altered signalings in *TMPRSS2:ERG* (TE) fusion positive prostate cancer.**

Other TE fusion positive and negative samples were obtained from The Cancer Genome Atlas (TCGA) data in purpose of validation. 2,983 genes in coordination with *ERG* in RNA expression (|R|>0.3) were identified and eight cancer-related pathways were identical with pathway observed in test set. One pathway, *p53*-independent G1/S DNA damage checkpoint, was not hooked up as altered pathway because of low incidence. 25% of genes among the genes associated with *p53*-independent G1/S DNA damage checkpoint pathway (2 genes out of 8 genes) were observed in validation set while 62.5% of genes (5 genes out of 8 genes) were found in test set.
