# Supplementary figures and images for: Comprehensive analysis of oncogenic signatures and consequent repurposed drugs in TMPRSS2:ERG fusion‐positive prostate cancer
Source: Clin Transl Med. 2021 May 13;11(5):e420. doi: 10.1002/ctm2.420 (PMC8120022; doi:10.1002/ctm2.420)

**Histogram of Rvals**

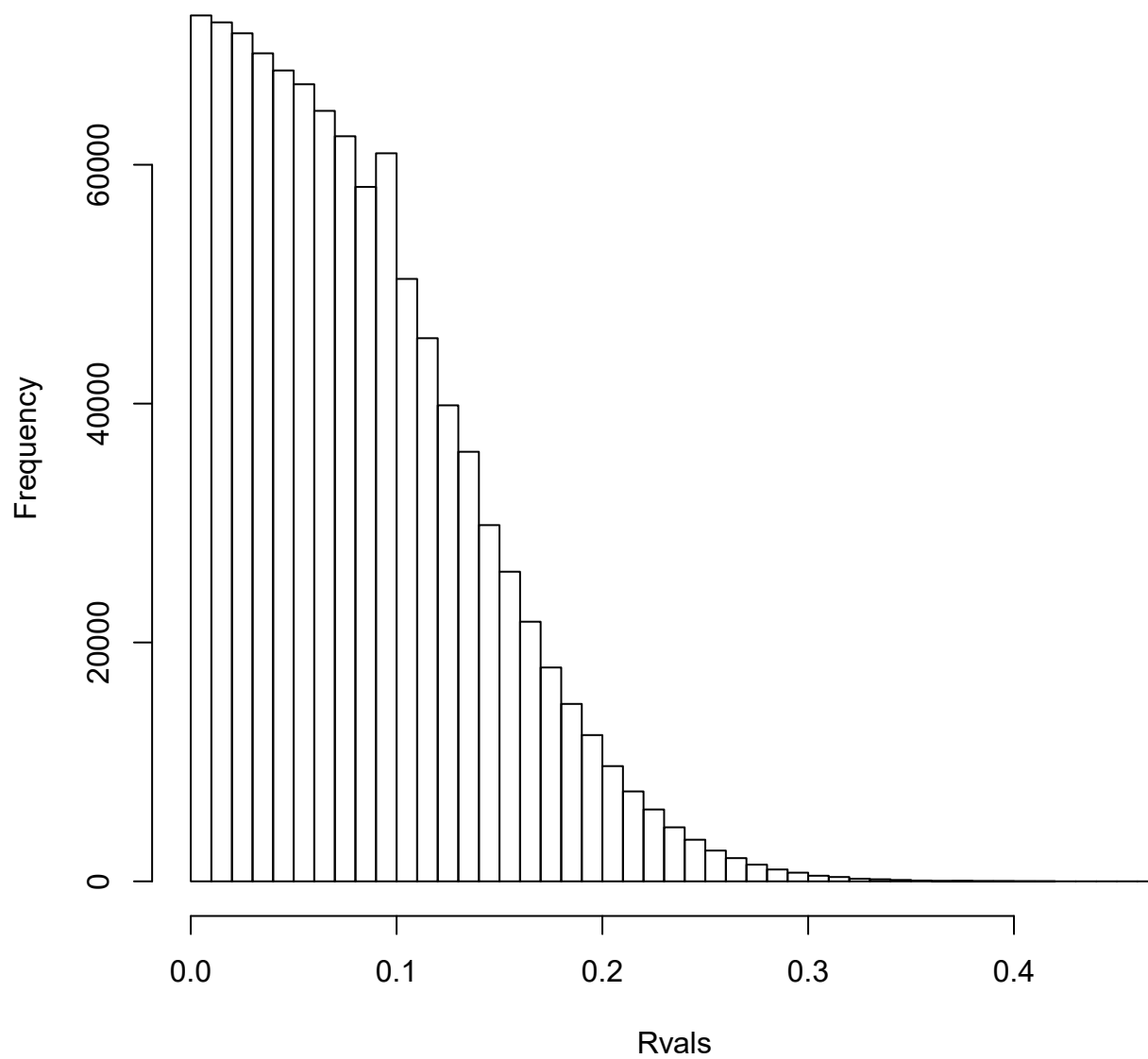

**Figure S1**

Supplement: Supplementary file 4 — Supporting information. [file CTM2-11-e420-s008.pdf]

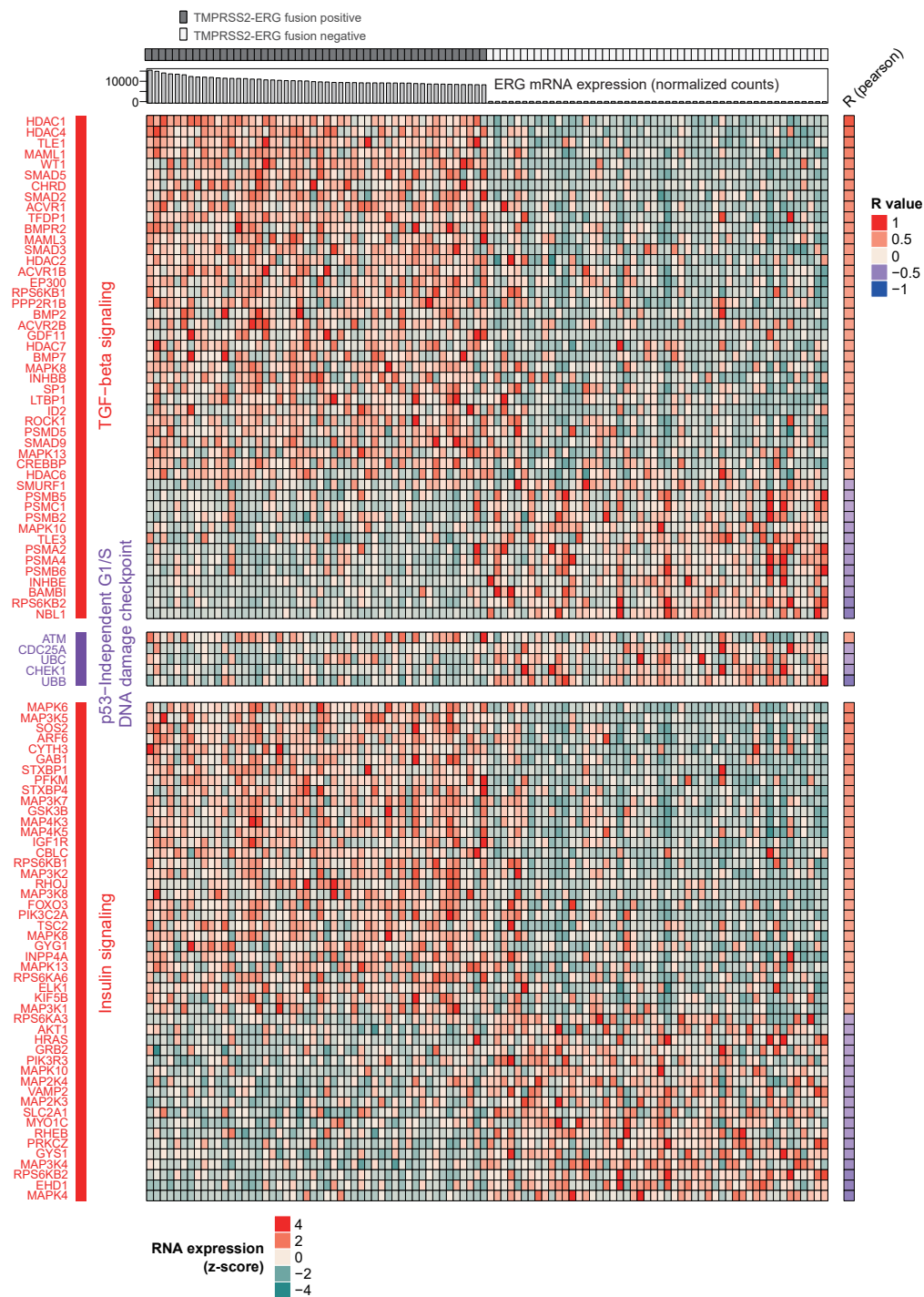

Figure S2

Supplement: Supplementary file 5 — Supporting information. [file CTM2-11-e420-s007.pdf]

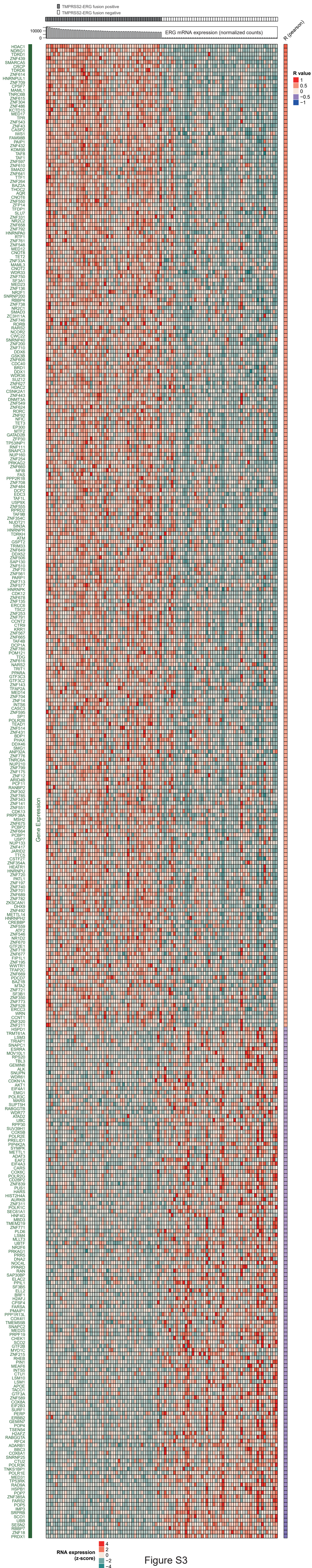

Figure S3

Supplement: Supplementary file 6 — Supporting information. [file CTM2-11-e420-s003.pdf]
