# Supplementary material for: Comprehensive analysis of oncogenic signatures and consequent repurposed drugs in TMPRSS2:ERG fusion‐positive prostate cancer
Source: Clin Transl Med. 2021 May 13;11(5):e420. doi: 10.1002/ctm2.420 (PMC8120022; doi:10.1002/ctm2.420)
Supplement: Supplementary file 8 — Supporting information. [file CTM2-11-e420-s001.pdf]

Pathway	Test set (p-value)	Validation set (p-value)
Androgen receptor Signaling	0.000318159	0.004526448
Gene expression Signaling	9.18E-13	1.55E-12
Insulin Signaling	0.000171896	0.020754181
NOTCH1 Signaling	0.00080743	0.006340186
p53 signaling	0.002220782	0.025577801
VEGFA-VEGFR2 Signaling	0.003186192	0.003220384
Wnt Signaling	0.000996148	0.003133151
TGF-beta Signaling	0.000257284	0.022056803

p53-independent G1/S DNA damage checkpoint pathway	Genelist
Ref	CDC25A, ATM, CHEK1, UBB, RPS27A, UBA52, UBC, CHEK2
Test set	CDC25A, ATM, CHEK1, UBB, UBC
Validation set	UBB, UBC

Figure S5
