# Supplementary material for: Comprehensive analysis of oncogenic signatures and consequent repurposed drugs in TMPRSS2:ERG fusion‐positive prostate cancer
Source: Clin Transl Med. 2021 May 13;11(5):e420. doi: 10.1002/ctm2.420 (PMC8120022; doi:10.1002/ctm2.420)
Supplement: Supplementary file 7 — Supporting information. [file CTM2-11-e420-s005.pdf]

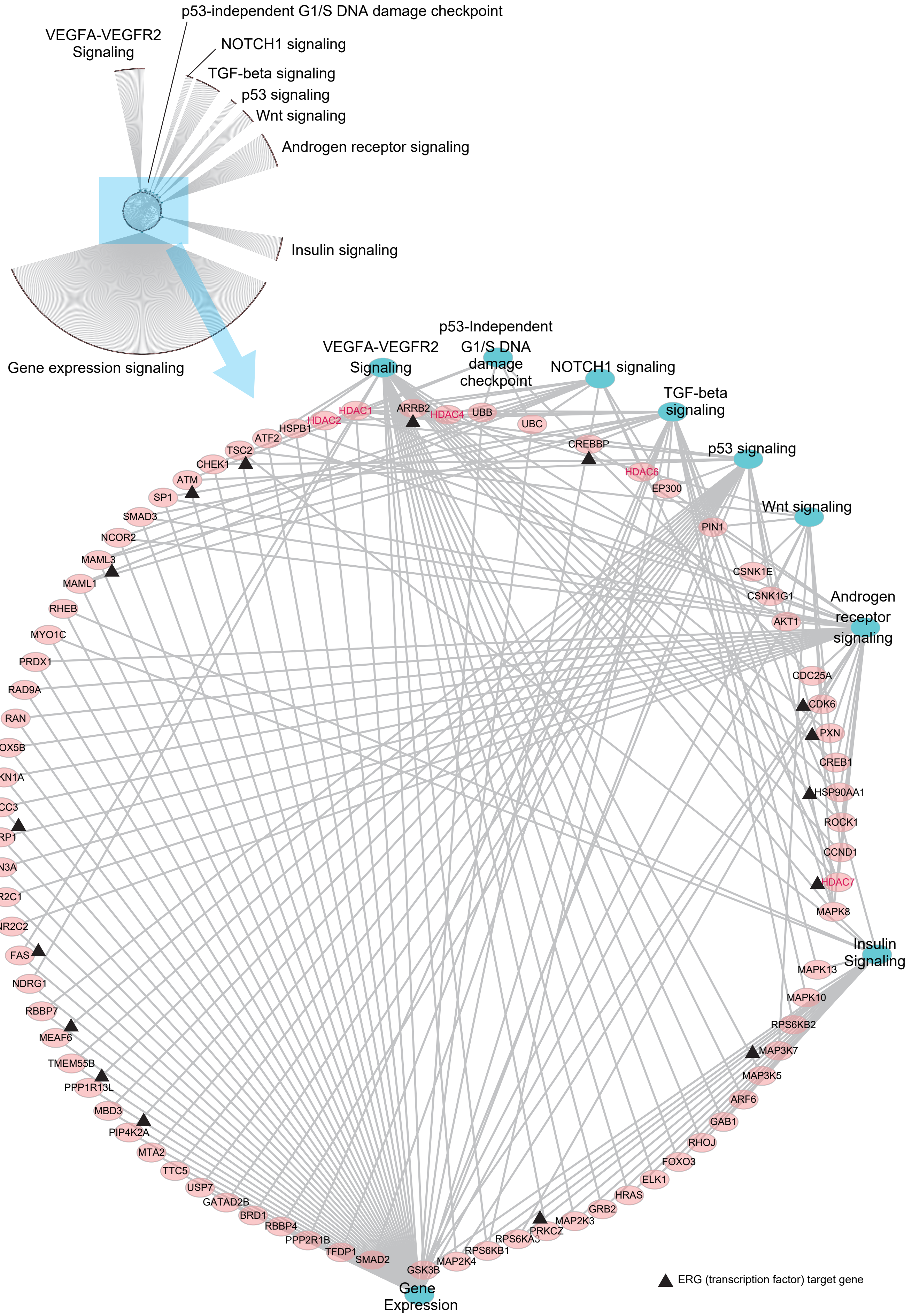
p53-independent G1/S DNA damage checkpoint
VEGFA-VEGFR2 Signaling
NOTCH1 signaling
TGF-beta signaling
p53 signaling
Wnt signaling
Androgen receptor signaling
Insulin signaling
Gene expression signaling
p53-Independent G1/S DNA damage checkpoint
VEGFA-VEGFR2 Signaling
NOTCH1 signaling
TGF-beta signaling
p53 signaling
Wnt signaling
Androgen receptor signaling
Insulin Signaling
Gene Expression
HDAC1
ARRB2
HDAC4
UBB
UBC
HDAC2
HSPB1
ATF2
TSC2
CHEK1
ATM
SP1
SMAD3
NCOR2
MAML3
MAML1
RHEB
MYO1C
PRDX1
RAD9A
RAN
COX5B
CDKN1A
ERCC3
PARP1
SIN3A
NR2C1
NR2C2
FAS
NDRG1
RBBP7
MEAF6
TMEM55B
PPP1R13L
MBD3
PIP4K2A
MTA2
TTC5
USP7
GATAD2B
BRD1
RBBP4
PPP2R1B
TFDP1
SMAD2
GSK3B
MAP2K4
RPS6KB1
RPS6KA3
PRKCZ
MAP2K3
GRB2
HRAS
ELK1
FOXO3
RHOJ
GAB1
ARF6
MAP3K5
MAP3K7
RPS6KB2
MAPK10
MAPK13
MAPK8
HDAC7
CCND1
ROCK1
HSP90AA1
CREB1
PXN
CDK6
CDC25A
AKT1
CSNK1G1
CSNK1E
PIN1
EP300
HDAC6
CREBBP
ERG (transcription factor) target gene

Figure S4
